# Changes in Mental State for Help-Seekers of Lifeline Australia’s Online Chat Service: Lexical Analysis Approach

**DOI:** 10.2196/63257

**Published:** 2025-06-20

**Authors:** Kelly Mazzer, Sonia Curll, Hakar Barzinjy, Roland Goecke, Mark Larsen, Philip J Batterham, Nickolai Titov, Debra Rickwood

**Affiliations:** 1 Faculty of Health, University of Canberra, 11 Kirinari Street, Bruce, Canberra, 2611, Australia, 610262015266; 2 School of Systems & Computing, University of New South Wales, Canberra, Australia; 3 Centre for Big Data Research in Health, University of New South Wales, Sydney, Australia; 4 Centre for Mental Health Research, Australian National University, Canberra, Australia; 5 School of Psychological Sciences, Macquarie University, Sydney, Australia

**Keywords:** crisis helpline, lexical analysis, mental health, outcomes, distress, affective computing, Lexical, suicidal, suicide, help-seeker, help-seeking, emotion, chat, mental state, caregivers, digital mental health, digital health, e-health, ANOVA, feasibility study, mental health intervention, crisis support, online communities, support service, online support

## Abstract

**Background:**

Mental health challenges are escalating globally, with increasing numbers of individuals accessing crisis helplines through various modalities. Despite this growing demand, there is limited understanding of how crisis helplines benefit help-seekers over the course of a conversation. Affective computing has the potential to transform this area of research, yet it remains relatively unexplored, partly due to the scarcity of available helpline data.

**Objective:**

This study aimed to explore the feasibility of using lexical analysis to track dynamic changes in the mental state of help-seekers during online chat conversations with a crisis helpline.

**Methods:**

Lexical analysis was conducted on 6618 deidentified online chat transcripts collected by Lifeline Australia between April and June 2023 using the validated Empath lexical categories of Positive Emotion, Negative Emotion, Suffering, and Optimism. Furthermore, 2 context-specific categories, Distress and Suicidality, were also developed and analyzed to reflect crisis support language. Correlation analyses evaluated the relationships between the 6 lexical categories. One-way ANOVAs assessed changes in each lexical category across 3 conversation phases (beginning, middle, and end). Trend analyses using regression modeling examined the direction and strength of changes in lexical categories across 9 overlapping conversation windows (20% size and 50% step overlap).

**Results:**

Significant changes were observed across conversation phases. The context-specific categories showed the strongest improvements from the beginning to end phase of conversation, with a large reduction in Distress (*d*=0.79) and a moderate reduction in Suicidality (*d*=0.49). The most frequently occurring terms representing Distress were “hard,” “bad,” and “down,” and for Suicidality were “suicide,” “stop,” and “hurt.” The negatively framed Empath categories also significantly reduced, with moderate effect sizes for Suffering (*d=*0.49) and Negative Emotion (*d=*0.39). There were also significant but small reductions in the positively framed Empath categories of Positive Emotion (*d=*0.15) and Optimism (*d=*0.07) from the beginning to end phase of conversation. Correlation coefficients indicated the lexical categories captured related but distinct constructs (*r*=.34 to *r*=0.82). Trend analyses revealed a consistent downward trajectory across most lexical categories. Distress showed the steepest decline (slope=−0.15, *R*²=0.97), followed by Suffering (slope=−0.11, *R*²=0.96), Negative Emotion (slope=−0.10, *R*²=0.69), and Suicidality (slope=−0.06, *R*²=0.88). Positive Emotion showed a slight negative trend (slope=−0.04, *R*²=0.54), while Optimism remained relatively stable across the conversation windows (slope=0.01, *R*²=0.13).

**Conclusions:**

This study demonstrates the feasibility of using lexical analysis to represent and monitor mental state changes during online crisis support interactions. The findings highlight the potential for integrating affective computing into crisis helplines to enhance service delivery and outcome measurement. Future research should focus on validating these findings and exploring how lexical analysis can be applied to improve real-time support to those in crisis.

## Introduction

### Background

Crisis helplines are a critical component of mental health care systems, offering immediate, confidential, free, and often 24/7 support to individuals experiencing emotional distress [1]. These services are delivered primarily via telephone, with many now offering text and web-based alternatives. Crisis supporters are usually staff members or volunteers trained in crisis and suicide intervention, with the skills and knowledge to provide support to help-seekers (also known as clients or users) and pathways to further care where needed. With the high prevalence of suicide deaths and emotional distress around the world, crisis helplines offer a cost-effective and scalable way to improve the accessibility and responsiveness of mental health and crisis care [1,2].

Crisis helplines face significant challenges in meeting the growing demand and diversity of help-seekers. Maintaining consistent, high-quality support across telephone and digital services is vital [2], especially as digital services are often used by vulnerable groups including youth and people with disabilities [3,4]. Crisis helplines also need to ensure they remain flexible and adapt quickly to the evolving communication preferences and needs of help-seekers, as highlighted during the recent COVID-19 pandemic [5]. These challenges, compounded by the urgency of crisis support, exacerbate the pressure on a crisis helpline’s resources and volunteer workforce [6-8].

Conducting research in crisis helpline settings poses multiple challenges, many of which stem from the anonymous and one-off nature of these services [2,9,10]. Most studies have relied on retrospective self-reports by help-seekers [11], which can provide valuable insight into subjective experiences but are subject to recall biases and fail to capture fluctuations in mental state during the contact. Moreover, low completion rates (eg, 33% completion rate among chatters in one study [3]) indicate potential self-selection bias, whereby help-seekers with more positive outcomes might be more likely to complete a postcontact survey. Alternative approaches, such as crisis supporter assessments, are limited by judgement biases (eg, social desirability concerns) and recall errors, while using external raters tends to be very labor-intensive with consequently small samples [12]. Improved approaches are urgently needed to inform strategies aimed at enhancing service delivery [2,4].

Integrating affective computing approaches within the crisis helpline context presents an opportunity for a transformative shift from traditional research approaches and holds substantial promise for enhancing mental health interventions [13]. Affective computing is concerned with developing systems and devices that can recognize, interpret, process, and simulate human emotions [14]. These systems collect and analyze various data on users’ mental states, including text-based emotional cues, vocal tone, and physiological signals. This information is then used by researchers and service providers to understand and enhance user experience in applications such as psychiatry, teaching, and social media [14].

In the helpline context, these advanced computational methods offer several ethical and practical advantages. Affective computing algorithms can be applied to large datasets, with the ability to detect meaningful patterns and insights difficult or impossible to obtain with traditional methods. Automated data collection techniques such as text or voice analysis can provide more representative and objective data than self-report of human-coded data by mitigating human judgment errors and biases (eg, recall, social desirability, and self-selection). They are also unobtrusive, imposing no additional burden on the help-seeker or crisis supporter. Crucially, affective computing techniques facilitate continuous assessment, opening possibilities for dynamic support tools and informing a deeper understanding of emotional responses and outcomes of accessing a crisis helpline [14].

Natural language processing (NLP) and computational linguistics play an important role in affective computing, especially in systems designed to process and understand emotions through text. These techniques can automatically analyze the words people use to provide insight into their mental states and emotions [15,16]. For instance, lexical analysis using existing and widely available lexicon-based software, such as Empath and Linguistic Inquiry and Word Count (LIWC) [17,18], has been applied to electronic health records to detect suicide risk [19] and to text-based transcripts from online therapy to predict depression symptom severity [20].

The past 5 years have seen a rapid growth in studies using NLP for mental health interventions [16,17] and it has now moved into the crisis helpline context, providing initial support for the development of NLP-based tools to provide adjunct assistance to crisis supporters. Recent research has demonstrated the usefulness of NLP to identify and classify self-harm or suicide risk among digital help-seekers [21,22]. Cognitive overload among digital crisis supporters has also been shown to reduce with support from NLP-based tools, including helping to resolve writer’s block and providing real-time information based on conversation content [23,24]. Furthermore, NLP can support the efficiency of crisis support, with Althoff et al [25] determining via NLP techniques that more successful online crisis support involved greater time devoted to exploring solutions, as opposed to defining problems.

However, few studies so far have applied NLP to understand help-seeker outcomes from crisis support. This is an important area to explore, as developing innovative ways to monitor and evaluate help-seeker outcomes is essential to maintaining quality service provision and informing service improvements [10,13]. Althoff et al [25] applied LIWIC to explore changes in help-seeker sentiment, being the relative proportion of positive to negative words, they demonstrated a trend toward a more positive perspective over the conversation, with a notable increase at the very end of the conversation. Progressing this area of research requires access to data from service providers, which necessitates sensitively and appropriately navigating protective regulations concerning data confidentiality and ethical considerations of help-seeker privacy [16,26].

### Current Study

This study is among the first to apply lexical analysis to explore changes in the frequency and intensity of language associated with mental states used by help-seekers over the duration of a single online chat conversation with a crisis helpline. Implementing lexical analysis overcomes many of the usual barriers to research in helplines. Specifically, it removes the need for human annotation, thereby eliminating the risks of annotator fatigue and bias, avoiding the resource heavy training of annotators, achieving greater efficiency and objectivity, and allowing for the analysis of a much larger amount of data [4,12,27]. This study aims to provide a proof-of-concept for using NLP to monitor the mental state of help-seekers. While some previous research has used NLP to explore broad changes in help-seekers’ positive and negative sentiment [eg, 25], in the current study we analyze a wider range of general mental states (Positive Emotion, Negative Emotion, Suffering, and Optimism) and crisis-specific mental states (Distress and Suicidality). In addition, our novel use of Empath allowed us to go beyond frequency (word counts) to examine context and reflect intensity, allowing a richer understanding of changes in help-seeker mental states. Our analyses examined trends across the crisis intervention including over 9 overlapping conversation windows as well as at the beginning, middle and end phases using a large deidentified dataset from Lifeline Australia’s online chat service.

## Methods

### Data

For more than 60 years, Lifeline has been operating as Australia’s national, free 24-hour telephone crisis support service. In recent years, Lifeline has also expanded its service delivery to also offer 24-hour digital support via text messaging and online chat services with crisis supporters [6]. Lifeline Australia provided a census of routinely collected data from all contacts made to their online chat service for 3 months from April to June 2023, totaling 20,569 contacts of varying lengths that were answered by a crisis supporter. Consistent with previous research [12], after excluding automated or chatbot messages, all conversations with 10 or more messages from the help-seeker were retained for analysis (N=6618). Data included the date of contact, time of each message, number of messages per conversation, and the content of messages from both the help-seeker and crisis supporter during the conversation. Help-seeker demographics were not available.

### Preprocessing

Lifeline Australia does not systematically collect identifiable information; however, any incidental identifying information contained in the messages were scrubbed before access and analysis. Data was divided into help-seeker or crisis supporter messages. Conversations were then split into equal thirds, based on total number of messages in the conversation, to create a beginning, middle, and end phase of each conversation. Conversations were also split into 9 overlapping conversation windows, using a 20% window size with a 50% step overlap, meaning that each subsequent window started at the midpoint of the previous window. Text was converted to lowercase and tokenized into individual words. The Porter Stemmer from The Natural Language Toolkit [28] was used to stem words by removing common morphological affixes and reducing words to their root forms, which enabled the capture of various word forms (“suicid*” to capture “suicide”, “suicidal”, “suicidality,” etc). In addition, n-grams, specifically bigrams, was used to capture meaningful word pairs (2-word phrases) as tokens [29], which provided a more accurate representation of the text’s semantic content (eg, “harm myself” as a single Suicidality term). All remaining messages from help-seekers were included in analysis; crisis supporter messages were not analyzed. Table 1 provides descriptive details of the dataset following preprocessing.

**Table 1.** Total counts, means, and SDs of help-seeker messages and lexical terms included in the final analyses for each online chat conversation (N=6618) and conversation phase (beginning, middle, and end).

Help-seeker data	Total count, n	Per conversation, mean (SD)	Per third^a^, mean (SD)
Messages	201,955	30.52 (20.55)	10.17 (6.86)
Terms^b^	309,628	46.79 (26.67)	15.60 (10.83)

a Thirds were created based on number of messages.

b Terms represent key words used in conversation, also known as tokens.

### Empath Software

Empath is an open-source Python (Python Software Foundation) library and text analysis tool that can perform NLP tasks including lexical analysis. Empath contains around 200 data-driven emotional and topical categories, also known as lexicons, which have been validated through a combination of NLP and human validation. Empath uses a large dataset to evaluate text and assign lexical degree scores based on the presence of predefined categories related to emotions, behaviors, and themes [17]. Empath lexicons are recognized as highly correlated to LIWC’s gold-standard categories (*r*=0.91) [17,18]. Each category has a large list of member terms (words) that represent the category; for example, the category of Optimism includes terms such as “hopeful,” “perseverance,” and “progress” [17]. Refer to Table 2 for the top 10 frequently occurring member terms for each category used in the current study.

**Table 2.** Characteristics of the lexical categories used in the analyses, including source, top 10 terms, total terms, total occurrences, and means and SDs of terms per conversation (N=6618).

Category	Empath or contextual	Top 10 terms^a^	Total terms^b^	Total occurrences^c^	Mean (SD) per conversation
Negative Emotion	Empath	Want, think, see, hard, bad, care, stop, hurt, scary or scar*, and die	94	73,008	11.03 (7.09)
Positive Emotion	Empath	Feel*, friend*, better, keep, family or famili*, care, love, understand, hope, and happi*	75	43,666	6.60 (4.57)
Suffering	Empath	Feel*, bad, hurt, die, long, depress*, wors*, kill, pain*, and cry or cri*	127	42,125	6.37 (4.28)
Optimism	Empath	Feel*, like, will, thank, sure, better, love, hope*, happy or happi*, and appreci*	81	47,442	7.17 (4.20)
Distress	Contextual	Hard, bad, down, hurt, scary or scar*, struggl*, alon*, depress*, stress*, and wors*	137	53,423	8.07 (5.71)
Suicidality	Contextual	Suicid*, stop*, hurt, die, kill, hate, pain*, plan, harm*, and safe*	163	31,428	4.75 (3.98)

a Top 10 terms represents 10 most frequently occurring terms in the dataset for each category, ranked in order of frequency.

b Total terms are the total number of member terms or words representing a category. Count does not include member terms where a single term represents the name of another Empath category. For example, Death is an Empath category as well as a member term of Suffering*;* all of Death’s 81 member terms would be identified as instances of Suffering*,* but are not included in the total terms value.

c Total occurrences are the total count of occurrences of member terms or words in conversations.

### Lexical Categories

#### Empath Categories

All validated Empath categories were reviewed and 4 were selected for inclusion as they best represented concepts that help-seekers may aim to improve by contacting a crisis helpline like Lifeline. The 4 categories were: Negative Emotion, Positive Emotion, Suffering, and Optimism. Lexical degree scores, ranging from 0 to 1, were assigned to text for the Empath categories of Negative Emotion, Positive Emotion, Suffering, and Optimism. A higher score represents a greater proportion of words present in the text that fall into a category. For example, a degree score of 0.7 for Suffering would indicate a very high occurrence of Suffering-related words in the conversation.

#### Contextual Categories

There is a risk that lexicons designed to understand general linguistic patterns, such as the Empath categories, may not be appropriate for highly domain specific tasks [30]. Thus, in addition to the 4 Empath categories, 2 new categories were derived for analysis. The categories of Distress and Suicidality are highly context specific; reducing distress and suicidality are 2 of the highest priority outcomes for help-seekers accessing Lifeline services [31].

Lexical categories representing Distress and Suicidality specific to the crisis helpline context were developed using a multistep process with input from various expert groups, including researchers, service providers, and people with lived experience. First, a team (n=8) of experienced crisis supporters from Lifeline Australia reviewed a sample of 200 online chat and text transcripts from a separate dataset not used in the current study. These crisis supporters received a training session from the research team (KM) on how to identify relevant keywords. They were also provided with written guidelines and examples to ensure consistency in their approach. The keywords identified by the crisis supporters were then collated and reviewed by the research team (KM and SC). The resulting list was then shared with several expert groups for further input and refinement, including Lifeline’s lived experience advisory group, online chat service team leaders, and Lifeline’s clinical practice team. The research team conducted a final review of all inputs, with any disagreements or ambiguous terms resolved through team discussion. Further refinement of keywords was undertaken using manual annotation of a random sample of 100 transcripts used in the current study (SC).

The same Empath lexical degree scoring system could not be applied for Distress and Suicidality as they were not pre-existing Empath categories. Instead, the lexical analysis of the contextual categories, Distress and Suicidality, were based on mean counts of terms present within the text. The mean of Distress represented the average number of occurrences of Distress member terms in a conversation.

The category of Distress had a total of 137 terms that occurred 53,423 times in the dataset; the top 10 Distress terms accounted for 40.26% (n=21,510) of these occurrences with “hard” being the most frequently occurring term (n=3118), followed by “bad” (n=2884), and “down” (n=2411).The Suicidality category had 163 total terms that occurred 31,428 times; the top 10 Suicidality terms accounted for 56.72% (n=17,825) of occurrences with “suicid*” occurring most frequently (n=2885), followed by “stop*” (n=2306), and “hurt” (n=2174).

### Statistical Analysis

**Figure 1.**
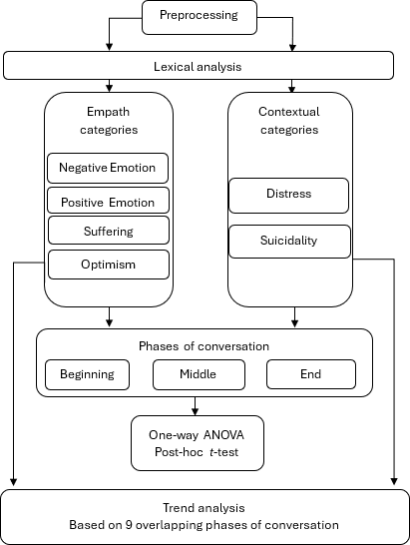
Overview of the design and structure of the study, including the lexical categories and conversation phases used in the analyses.

Pearson correlations were conducted based on the number of term occurrences to determine the strength of relationship between the 6 lexical categories. Lexical analyses were conducted to examine changes in mental state over the phases (beginning, middle, and end) of an online chat conversation with a help-seeker. One-way ANOVA was performed to determine whether the degree of occurrence of each category was significantly different between the start, middle, and end phases of conversation. Post hoc *t* tests were also conducted to determine which phases of conversation were significantly different from one another. Trend analysis and linear regression were then performed on the mean token counts across 9 overlapping conversation windows to determine the direction (slope) and magnitude (*R*²) of change within each lexical category. This method allowed better understanding of the dynamic change across conversations. Figure 1 shows the workflow of analyses and included categories.

### Ethical Considerations

This study was approved by the University of Canberra’s Human Research Ethics Committee (approval no. 4673). The data that were analyzed were collected routinely by Lifeline Australia. Due to the anonymity of Lifeline Australia help-seekers, there was no opportunity to obtain specific consent from individuals to use their data. However, all Lifeline Australia help-seekers are informed that their personal data may be used to conduct research, evaluation, and assurance activities. No identifying information (eg, phone number, email address, or help-seeker name) were provided in the dataset. Deidentified data were stored and analyzed in a secure, Lifeline owned and managed environment.

## Results

### Overview

Descriptive statistics of conversation length are presented, followed by correlations between all categories. Results for each of the Empath categories of Negative Emotion, Positive Emotion, Suffering and Optimism, are presented as means and SD of lexical degree scores for all conversations separated into the beginning, middle, and end phases of conversation. The contextual categories of Distress and Suicidality are presented as mean occurrences of terms across all conversations in the beginning, middle, and end phases. Tests of significance are reported for changes in each category over the phases of conversation.

### Descriptive Statistics

Conversations included in the final analyses had a range of 10 to 382 help-seeker messages (mean 30.52, SD 20.55; Table 1). A total of 309,628 occurrences of terms from all included lexical categories were identified, with a mean of 46.79 (SD 26.67) occurrences of terms per conversation. Negative Emotion had the highest occurrences of terms at 73,008, followed by Distress with 53,423. Note that categories can and do include overlapping member terms; in fact, similarity comparisons are used in Empath’s mapping of vocabulary to categories [17]. “Feel*” (feel, feels, feeling, and feelings) was among the most frequently occurring words for many of the emotion-based categories. Table 2 provides descriptions of the categories and their occurrence at the conversation level.

### Correlations

Table 3 presents the correlations between mean number of occurrences across the categories. Positive Emotion and Optimism had the strongest relationship (*r*=0.82), whereby when a help-seeker expresses positive emotions they are also likely to use optimistic language. The next strongest correlations were between Distress with both Suffering (*r*=0.81) and Negative Emotion (*r*=0.77). These indicate that help-seekers experiencing high level of distress are also likely to express a lot of suffering and negative emotion. Correlations between all categories revealed significant, *P*<.001, medium, or strong positive relationships (*r*=0.34 to 0.82), likely reflecting the shared foundation of emotion and emotion-related terms that each of these categories is defined by. No categories had correlations higher than *r=*0.82, suggesting each category represented a distinct construct.

**Table 3.** A correlation matrix showing the relationships between mean lexical category occurrences in help-seeker online chat messages.

Lexical category^a^	1	2	3	4	5
Negative Emotion					
Positive Emotion	.71				
Suffering	.63	.68			
Optimism	.63	.82	.63		
Distress	.77	.67	.81	.64	
Suicidality	.62	.38	.58	.34	.45

a *P*<.001.

### Changes in Emotions by Phase of Conversation

**Figure 2.**
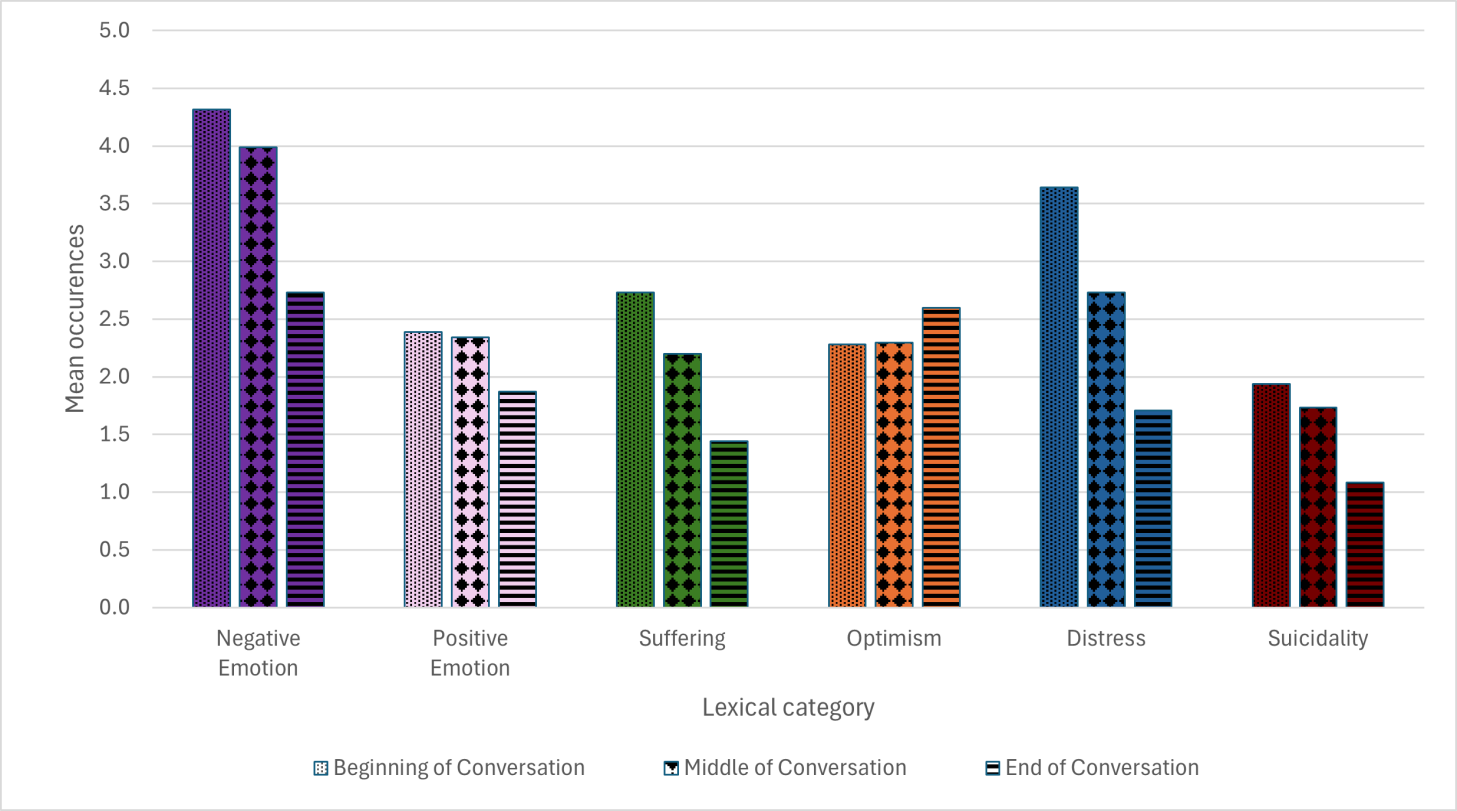
Mean occurrences of lexical category terms across 3 phases of help-seeker online chat conversation (beginning, middle, and end).

Figure 2 presents the mean occurrence of terms by category for the beginning, middle, and end phases of the conversation. Except for Optimism, all categories revealed a pattern of reduction in the number of term occurrences from the beginning phase of conversation to the end. Surprisingly, this includes the positively framed category of Positive Emotion*,* which would be expected to increase during a contact with Lifeline’s online chat service.

### Lexical Analyses

#### Empath Categories by Conversation Phase

Table 4 presents the lexical degree scores for all 4 Empath categories by phase of conversation. Help-seekers most frequently used terms related to Negative Emotion across all 3 phases. Both negative categories, Negative Emotion (*F*_19851,2_=273.68, *P*<.001) and Suffering (*F*_19851,2_=424.30, *P*<.001) significantly improved from the beginning, to middle, to end phase of conversation with both demonstrating medium effect sizes from beginning to end of conversation (*d*=0.39 and *d*=0.49, respectively). Surprisingly, Positive Emotion (*F*_19851,2_=54.42, *P*<.001) and Optimism (*F*_19851,2_=70.49, *P*<.001) also significantly reduced in the end phase of conversation compared to the beginning, although only with weak effect sizes (*d*=0.15 and *d*=0.07, respectively). Note that the pattern of results based on mean occurrence was similar to the pattern of results based on mean lexical degree score for all categories except Optimism (Figure 2).

**Table 4.** One-way ANOVA and post hoc *t* tests comparing lexical degree scores across conversation phases (beginning, middle, and end) for Empath lexical categories.

Lexical category	*F* test (*df*)	Mean lexical degree score (SD)^b^			Cohen *d*		
		Beginning	Middle	End	Beginning-middle	Middle-end	Beginning-end
Negative Emotion	273.68^a^ (19851,2)	0.0248^b^ (0.0224)	0.0228^c^ (0.0210)	0.0166^d^ (0.0198)	0.09	0.31	0.39
Positive Emotion	54.42^a^ (19851,2)	0.0129^b^(0.0153)	0.0107^c^ (0.0130)	0.0106^c^ (0.0147)	0.15	0.01	0.15
Suffering	424.30^a^ (19851,2)	0.0134^b^ (0.0162)	0.0090^c^ (0.0131)	0.0065^d^ (0.0118)	0.30	0.20	0.49
Optimism	70.49^a^ (19851,2)	0.0077^b^ (0.0121)	0.0054^c^ (0.0094)	0.0068^d^ (0.0120)	0.21	–0.13	0.07

a *P*<.001.

b Differing (b,c,d) superscripts represents differences at *P*<.001 between conversation phases. Where the same superscript is shown across row (b,b), the conversation phases did not differ significantly.

c Differing (b,c,d) superscripts represents differences at *P*<.001 between conversation phases. Where the same superscript is shown (c,c), the conversation phases did not differ significantly.

d Differing (b,c,d) superscripts represents differences at *P*<.001 between conversation phases.

#### Contextual Categories by Conversation Phase

Table 5 presents changes in Distress and Suicidality based on the mean number of occurrences of terms. Both contextual categories significantly improved from the beginning, to middle, to end phase of conversation (Distress *F*_19851,2_=1338.07, *P*<.001 and Suicidality *F*_19851,2_=421.45, *P*<.001). Distress had the strongest effect size of any category (Cohen *d*=0.79), in its reduction from the beginning to the end of the conversation. Suicidality was also among the strongest effects of all categories with Cohen *d*=0.49 from the beginning to the end of the conversation.

**Table 5.** One-way ANOVA and post hoc *t* tests comparing mean number of occurrences across conversation phases (beginning, middle, and end) for contextual lexical categories.

Category	*F*^a^ test (*df*)	Mean (SD)^b^			Cohen *d*		
		Beginning	Middle	End	Beginning-middle	Middle-end	Beginning-end
Distress	1338.07^a^ (19851,2)	3.64^b^ (2.89)	2.73^c^ (2.45)	1.70^d^ (1.93)	0.34	0.47	0.79
Suicidality	421.45^a^ (19851,2)	1.93^b^ (1.98)	1.73^c^ (1.81)	1.08^d^ (1.46)	0.11	0.40	0.49

a *P*<.001.

b Differing (b,c,d) superscripts represents differences at *P*<.001 between conversation phases. Where the same superscript is shown acros row (b,b), the conversation phases did not differ significantly.

c Differing (b,c,d) superscripts represents differences at *P*<.001 between conversation phases.

d Differing (b,c,d) superscripts represents differences at *P*<.001 between conversation phases.

### Trend Analyses

**Figure 3.**
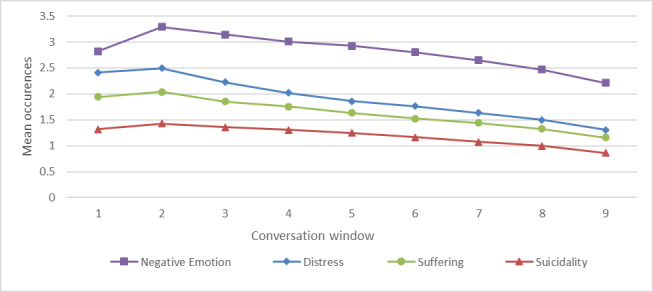
Trend of mean occurrences for negative lexical categories (Negative Emotion, Distress, Suffering, Suicidality) across 9 overlapping conversation windows.

Regression results indicated a consistent downward trajectory for Distress (slope=−0.1471, *R*²=0.9741), Suicidality (slope=−0.0636, *R*²=0.8821), Negative Emotion (slope=−0.1017, *R*²=0.6937), and Suffering (slope=−0.1053, *R*²=0.9644), suggesting a steady decline in the expression of these categories over the course of the conversations (Figure 3). Among these, Distress maintained the steepest decline, aligning with findings from previous analyses demonstrating significant reductions in distress-related language.

**Figure 4.**
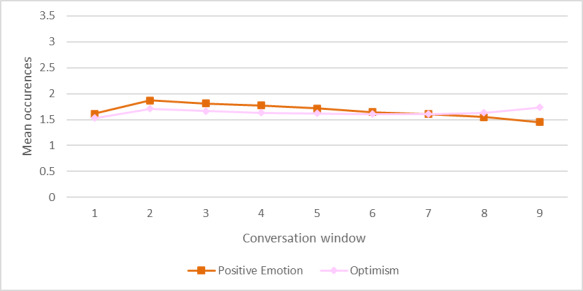
Trend of mean occurrences for positive lexical categories (Positive Emotion, and Optimism) across 9 overlapping conversation windows.

Positive Emotion also displayed a slight negative trend (slope=−0.0359, *R*²=0.5372), but its decline was less pronounced compared with the negatively framed categories. On the other hand, optimism remained relatively stable, with a weak positive slope (slope=0.0080, *R*²=0.1253), which indicates minimal variation across the conversation windows (Figure 4).

The strength of the regression models varied across categories. Distress, Suicidality, and Suffering, had high *R*² values, indicating strong model fit and predictable changes over time. In contrast, Optimism had the lowest explanatory power, suggesting that its variations may be influenced by additional conversational factors not captured by the linear model. Table S1 in Multimedia Appendix 1 provides means and SD for each category across the 9 windows.

## Discussion

### Principal Findings

In this study, lexical analysis was combined with traditional statistical techniques to detect changes in language associated with the mental state of help-seekers accessing a chat-based national crisis helpline. Results provide meaningful new insights into how people interact with crisis chat helplines. Crucially, this research is the first to demonstrate the feasibility of a novel methodological approach that may facilitate unobtrusive, objective, and real-time assessment of help-seeker outcomes, with the potential to enhance the effectiveness and efficiency of crisis helplines globally.

Use of negative language reduced across each phase of the crisis chat conversation, from beginning, middle, to end. This pattern was evident across all categories representing negative mental states (Negative Emotion, Suffering, Distress, and Suicidality). Trend analyses across 9 overlapping conversation windows showed a small increase in use of negative language from the first to second window, followed by decreases across the remaining windows. These findings are consistent with meta-analytical evidence for the overall effectiveness of crisis helplines in reducing emotional distress and risk of suicide [9,13], providing validation for our approach. Specific to the chat modality, previous evidence based on help-seeker self-report has found that experiences of distress and feeling suicidal reduced pre-post contact [3]. The current findings strengthen support for this outcome by demonstrating a similar decline in distress and suicidality during the conversation, using an approach unaffected by selection bias (ie, that only help-seekers who felt better or less suicidal completed the postcontact measures). Reducing distress is a core pillar of crisis helplines and has been identified as the most important outcome for help-seekers [31]. Distress had the largest reduction over the conversations in the current study, showing a welcome marker of the effectiveness of crisis helplines.

The reduction in suicidality-related words is also encouraging and aligns with suicide prevention as a key objective of service delivery for crisis helplines [31]. More than 90% of conversations in the current study involved suicidality-related content. Disclosures and discussions about suicide in a helpline context can occur at different stages of the contact depending on the needs of the help-seeker. While any immediate risk of suicide is addressed as soon as it is identified (beginning stage), for other help-seekers the sensitivity of the issue means it may be better discussed after rapport has been established (middle stage) [32]. That the largest reduction in suicidality-related words occurred in the end phase of the conversation in this study supports this notion. The trend analysis further confirms this finding, as Suicidality maintained a downward trajectory across the conversation windows, indicating that the largest reduction in suicidality-related words occurred toward the later stages of the conversation. However, it is important to consider that these aggregate results mask individual variations. The timing of disclosure might also vary between telephone versus digital services, with previous research suggesting greater digital disclosure of sensitive and stigmatized issues among youth [33]. Future studies exploring typical patterns in how and when suicidality is disclosed and discussed across different modalities are needed.

Contrary to expectations, there was a trend toward less expression of positive language across the conversation. Positive Emotion was higher in the beginning, compared with the middle and end phases, although these effects were weak. Findings based on lexical degree scores indicated that optimism was also highest in the beginning, although average word use was highest in the end phase of the conversation. Trend analyses across 9 overlapping segments also showed a slight decline in Positive Emotion, while Optimism was relatively stable. Unlike simple word occurrence counts, lexical degree scores account for term associations and strengths within a category, and hence these approaches can show some, though not substantive, divergence of results across conversation phases. Rather than indicating ineffectiveness, we suggest the heightened initial positive mental state may reflect hope or relief from deciding to seek help. In addition, the crisis supporter’s model of practice is to, first, establish a connection with the help-seeker and then, second, explore their concerns, at which stage an understandable reduction in positive language may occur. Moreover, previous research indicates that most help-seekers do not access crisis support to achieve a positive emotional state but to alleviate an intensely negative one [31,34]. We suggest that while tracking positive mental states may be useful for understanding the complex dynamics of crisis helpline conversations, care should be taken when using positive emotions as outcomes or an indicator of service effectiveness.

### Implications for Research and Practice

This proof-of-concept study answers recent calls for innovative approaches to data collection in the crisis helpline context [10,13,25]. The capacity of lexical analysis and advanced affective computing approaches to automatically detect and analyze emotion-based language in large datasets (and potentially in real-time) has the potential to transform crisis helpline research. Affective computing approaches hold promise for improved training instruments, quality assessment, and tools to help crisis supporters understand and respond to help-seekers’ needs.

The practical implications of our findings are considerable. By tracking help-seeker mental states in real-time, lexical analysis–based tools may be able to enhance crisis supporter responsiveness, through methods such as visual aids or dashboards that support risk assessment and emotion detection. Such tools might be especially useful in the text and chat contexts where emotion recognition is more challenging due to the absence of vocal cues [35,36]. Similar tools could also be used for professional development and to enhance crisis supporter motivation and satisfaction, thus improving volunteer retention and reducing the risk and impact of negative well-being and burnout. Stronger evidence for crisis helpline effectiveness will facilitate funding opportunities and support more tailored service delivery.

The current findings were based on online chat data and other modalities, like telephone or SMS text message services, may show a different pattern of results. For example, some research shows that help-seekers accessing online crisis helplines have higher levels of suicidality than those accessing telephone helplines [37]. As such, it will be important for future studies to conduct comparative analyses across modalities and to cross-validate NLP methods for monitoring user outcomes with real-time data.

The potential real-time application of lexical analysis-based tools in crisis helplines raises practical and ethical challenges that warrant careful consideration [38,39]. From a practical perspective, ongoing validation of context-specific categories with real-world data will be critical to maintaining accuracy and responsiveness. Language evolves rapidly, particularly in digital environments, with new slang, cultural references, and mental health terminology varying across social contexts [30]. Future research could explore the development of automated methods for continuous learning to detect and incorporate new keywords, enhancing the adaptability of these tools. Ethically, there is a risk of over-reliance on automated tools at the expense of human judgment, potentially comprising the quality of crisis support. Furthermore, the use of these tools must be balanced with the paramount importance of help-seeker confidentiality and privacy. To navigate these ethical challenges, crisis helplines could consider implementing informed consent processes or opt-in mechanisms to provide users with choice and control over the use of artificial intelligence–assisted tools in their interactions. To ensure transparency and maintain the trust of help-seekers, crisis supporters, and the broader community, crisis helplines should develop and publish clear policies on the role of artificial intelligence in crisis interventions, detailing how these tools are used, what data is collected, and how it is protected. Regular evaluation of the risks and benefits, together with robust safeguarding procedures, can help ensure new tools enhance rather than compromise the quality and ethics of crisis intervention delivery. As this field of research grows, it is vital to develop frameworks that ensure research rigor and integrity, including ways to facilitate the safe and ethical sharing of highly sensitive data between service providers and researchers [2,10,40].

### Limitations

This investigation revealed several interesting patterns in crisis chat conversations. The use of both pre-existing Empath categories alongside newly developed, context-specific categories, which both showed a similar pattern of results, strengthens the reliability of our findings [41]. However, there are important limitations to note.

A well-known limitation of lexicon-based approaches is their inability to account for context (contextual agnosticism). For example, the use of the word suicide in a chat does not indicate whether the help-seeker is talking about suicide generally or expressing their own suicidality. Similarly, this approach is unable to account for sarcasm, negations, or misspellings. This deficit may be particularly problematic when analyzing crisis online chat conversations, where the language used is often fragmented and ambiguous [4], and may have additional variations associated with computer-mediated communication norms (eg, emojis and abbreviations). To help overcome this limitation, future research could integrate lexical analysis with other approaches, such as sentiment analysis, topic modeling, and deep-learning features such as contextualized embeddings (eg, RoBERTa) [42]. However, more complex approaches would sacrifice the transparency and interpretability of lexicon-based methods [16,43].

Another limitation of lexical-based approaches is the correlation within categories, that is, the overlap between different but related emotional categories. Specifically, the same lexical term, “feel”, represented the top occurring term in multiple categories (Positive Emotion, Suffering, and Optimism). This overlap may hide nuanced shifts in specific mental states, with patterns across categories appearing similar due to their shared expressions [43]. Notably, correlations between the categories included in the current formative study were not exceedingly high and indicated sufficient variance to represent different constructs. Moreover, some overlap in terms and language used to express various emotions is to be expected. Future research may benefit from exploring those relationships between emotions using an approach such as network analysis.

Furthermore, data limitations constrain the generalizability of our findings. Analyzing data from a single helpline (Lifeline Australia), modality (online chat), and time period (3 months) increases risks of bias [44]. A common challenge in crisis helpline research is the lack of access to demographic information about help-seekers due to the anonymous nature of service engagement, which limits understanding of variations across groups [5]. Thus, it remains unclear whether the changes in mental states identified in this study would generalize to other helpline settings, temporal contexts, or service modalities.

Our formative approach of dividing the chats into thirds based on the total number of messages, as well as 9 overlapping windows, in the conversation used 2 methods to enhance comparability across chats of varying pace and length and allowed sufficient data in each segment for meaningful analysis. The use of 9 overlapping windows provides a more nuanced understanding of lexical shifts as it captures subtle fluctuations that may be obscured when dividing conversations into discrete phases (as in beginning, middle, and end). However, it is important to acknowledge that the unique and dynamic nature of each help-seeking process may not be entirely captured by the quantity of text exchanged. Future research may benefit from a dynamic and integrated approach that combines timestamps with message counts to identify more meaningful conversation stages. This could potentially capture both the temporal aspects and content progression of crisis chat interactions.

Finally, our approach assumes that the words used by help-seekers in the conversations reflect key help-seeker outcomes (changes in mental state and suicide risk). While this is likely the case, the validity of using lexical analysis to measure such outcomes needs to be further tested. For example, studies could compare the results of automated text analysis to expert human coding of the same conversations to determine concordance, or triangulate lexical results with self-report measures.

### Conclusions

A large dataset of crisis chats from Australia’s national helpline demonstrated how NLP techniques can be used to track language associated with help-seeker mental states. Pending positive results from future validation studies, lexical analysis has the potential to be a valuable tool in monitoring and evaluating outcomes for help-seekers accessing a crisis chat service. The findings should be viewed as a successful test for the feasibility of approach rather than a real-world tool. We hope this formative research and initial step encourages further research toward the development and implementation of tools that can help crisis helplines meet the expanding needs of help-seekers in crisis.

## Supplementary material

10.2196/63257 Multimedia Appendix 1 Means and SDs of token counts for each category across the 9 windows.

## Abbreviations

LIWC: Linguistic Inquiry and Word Count
NLP: natural language processing
