# Supplementary material for: Changes in Mental State for Help-Seekers of Lifeline Australia’s Online Chat Service: Lexical Analysis Approach
Source: JMIR Form Res. 2025 Jun 20;9:e63257. doi: 10.2196/63257 (PMC12204239; doi:10.2196/63257)
Supplement: Multimedia Appendix 1 [file formative-v9-e63257-s001.docx]

Table S1. Means and standard deviations of token counts for each category across the nine windows.

| Lexical Category | Window 1 | | Window 2 | | Window 3 | | Window 4 | | Window 5 | | Window 6 | | Window 7 | | Window 8 | | Window 9 | |
| --- | --- | --- | --- | --- | --- | --- | --- | --- | --- | --- | --- | --- | --- | --- | --- | --- | --- | --- |
|  | M | SD | M | SD | M | SD | M | SD | M | SD | M | SD | M | SD | M | SD | M | SD |
| Distress | 2.41 | 2.22 | 2.50 | 2.18 | 2.22 | 2.09 | 2.02 | 1.97 | 1.86 | 1.87 | 1.76 | 1.79 | 1.64 | 1.73 | 1.50 | 1.63 | 1.31 | 1.51 |
| Suicidality | 1.32 | 1.61 | 1.43 | 1.59 | 1.36 | 1.54 | 1.31 | 1.52 | 1.25 | 1.46 | 1.17 | 1.38 | 1.08 | 1.35 | 1.00 | 1.32 | 0.86 | 1.23 |
| Suffering | 1.94 | 1.69 | 2.04 | 1.68 | 1.85 | 1.63 | 1.76 | 1.64 | 1.63 | 1.56 | 1.52 | 1.49 | 1.44 | 1.49 | 1.32 | 1.44 | 1.16 | 1.34 |
| Negative Emotion | 2.82 | 1.52 | 3.29 | 1.65 | 3.14 | 1.65 | 3.01 | 1.66 | 2.92 | 1.63 | 2.80 | 1.60 | 2.65 | 1.55 | 2.47 | 1.57 | 2.21 | 1.53 |
| Optimism | 1.53 | 1.31 | 1.71 | 1.36 | 1.66 | 1.38 | 1.63 | 1.37 | 1.62 | 1.38 | 1.61 | 1.38 | 1.61 | 1.41 | 1.63 | 1.42 | 1.74 | 1.48 |
| Positive Emotion | 1.61 | 2.54 | 1.87 | 2.62 | 1.81 | 2.54 | 1.77 | 2.49 | 1.71 | 2.47 | 1.64 | 2.34 | 1.60 | 2.24 | 1.55 | 2.21 | 1.45 | 2.11 |
